# Pathophysiology and management of classic galactosemic primary ovarian insufficiency

**DOI:** 10.1530/RAF-21-0014

**Published:** 2021-06-25

**Authors:** Synneva Hagen-Lillevik, John S Rushing, Leslie Appiah, Nicola Longo, Ashley Andrews, Kent Lai, Joshua Johnson

**Affiliations:** 1 Department of Pediatrics, University of Utah School of Medicine, Salt Lake City, Utah, USA; 2 Department of Nutrition and Integrative Physiology, University of Utah College of Health, Salt Lake City, Utah, USA; 3 Divisions of Reproductive Sciences, Reproductive Endocrinology and Infertility, Department of Obstetrics and Gynecology, University of Colorado Denver (AMC), Aurora, Colorado, USA; 4 Division of General Obstetrics and Gynecology, Department of Obstetrics and Gynecology, University of Colorado Denver (AMC), Anschutz Outpatient Pavilion, Aurora, Colorado, USA

**Keywords:** primary ovarian insufficiency, ovary, follicle, reproductive endocrinology, fertility preservation

## Abstract

Classic galactosemia is an inborn error of carbohydrate metabolism associated with early-onset primary ovarian insufficiency (POI) in young women. Our understanding of the consequences of galactosemia upon fertility and fecundity of affected women is expanding, but there are important remaining gaps in our knowledge and tools for its management, and a need for continued dialog so that the special features of the condition can be better managed. Here, we review galactosemic POI and its reproductive endocrinological clinical sequelae and summarize current best clinical practices for its management. Special consideration is given to the very early-onset nature of the condition in the pediatric/adolescent patient. Afterward, we summarize our current understanding of the reproductive pathophysiology of galactosemia, including the potential action of toxic galactose metabolites upon the ovary. Our work establishing that ovarian cellular stress reminiscent of endoplasmic reticulum (ER) stress is present in a mouse model of galactosemia, as well as work by other groups, are summarized.

**Lay summary:**

Patients with the condition of classic galactosemia need to maintain a strict lifelong diet that excludes the sugar galactose. This is due to having mutations in enzymes that process galactose, resulting in the buildup of toxic metabolic by-products of the sugar. Young women with classic galactosemia often lose the function of their ovaries very early in life (termed 'primary ovarian insufficiency'), despite adherence to a galactose-restricted diet. This means that in addition to the consequences of the disease, these women also face infertility and the potential need for hormone replacement therapy. This article summarizes current strategies for managing the care of galactosemic girls and women and also what is known of how the condition leads to early primary ovarian insufficiency.

## Introduction

Classic galactosemia is an inborn error of carbohydrate metabolism associated with early-onset primary ovarian insufficiency (POI) in young women. Our understanding of the consequences of galactosemia upon fertility and fecundity of affected women is expanding, but there are important remaining gaps in our knowledge and tools for its management. For example, while clinical consensus is building about how best to manage patients with galactosemia in prepubertal life and the reproductive years, there is a general lack of established ‘best practice’ strategies. These gaps also include open questions about the mechanism(s) by which galactosemic metabolic perturbations impact reproductive organs, in particular, the ovary. Indeed, systemic production of ‘toxic metabolites’ of galactosemia is often referenced as a causal factor in compromised physiology generally, but direct evidence that individual metabolites can induce ovarian damage has been elusive. In this review, we attempt to fill in some of these gaps. First, we review the condition and its reproductive endocrinological clinical sequelae and summarize current best clinical practices for the management of this quite severe, early-onset POI. Afterward, we set out the state of our understanding of the reproductive pathophysiology of galactosemia, including the potential action of toxic galactosemic metabolites on the ovary. Our work establishing that ovarian cellular stress reminiscent of endoplasmic reticulum (ER) stress is present in a mouse model of galactosemia, as well as work by other groups, are summarized in this latter review section.

## Classic galactosemia

Galactosemia results in the deficiency of enzymes in the Leloir pathway (Holden *et al.* 2003) including galactokinase (GALK1), galactose-1-phosphate uridylyltransferase (GALT), galactose mutarotase (GALM), or UDP-galactose 4′-epimerase (GALE), any of which can compromise the metabolism of galactose (Wada *et al.* 2019). The most common and severe form of galactosemia is GALT deficiency, also called classic galactosemia (CG), and affects 1/14,000–1/80,000 individuals worldwide (Jumbo-Lucioni *et al.* 2012) and 1:53,554 births in the United States of America (Therrell *et al.* 2014). CG is inherited in an autosomal recessive pattern. Newborns in many countries are screened through screening programs for CG after birth by measuring GALT enzyme activity or galactose in dried blood spots. Infants that screen positive have further diagnostic testing to allow a timely diagnosis early in life. Diagnosis of CG includes detection of reduced GALT enzyme activity in red blood cells, elevated erythrocyte galactose-1-phosphate (gal-1P) levels, and, in general, the identification of pathogenic variants in the *GALT* gene (Pyhtila *et al.* 2015). Initial symptoms for infants include emesis and poor feeding with failure to thrive, lethargy, jaundice, liver failure, and Gram-negative sepsis. Infants who survive the acute presentation or are treated early because of newborn screening still suffer chronic long-term complications including developmental delay, speech and language delays, motor function abnormalities, cataracts, absent or delayed puberty or premature menopause as a result of ovarian insufficiency (Fridovich-Keil *et al.* 2011, Berry 2020).

Treatment for CG includes a galactose-restricted diet to avoid exposure to sugar. This results in the reduction, but not in the normalization of metabolites that accumulate inappropriately due to the deficiency of the GALT enzyme. The metabolites galactitol and gal-1P are known to have toxic effects upon cells and are thought to contribute to CG pathophysiology (Thakur *et al.* 2017). Dietary galactose restriction resolves initial neonatal symptoms, but chronic complications persist with varying prevalence and severity despite adherence to dietary management. Although a galactose-restricted diet is started as soon as the diagnosis is confirmed, the exact timing of diet initiation does not have long-term effects on the development of chronic conditions later in life (Jumbo-Lucioni *et al.* 2012).

The persistent elevation of galactitol and gal-1P is likely due to the endogenous galactose production from UDP-galactose via UPD-glucose, which results in elevated levels of galactose, galactitol, and gal-1P in CG patients despite being on a galactose-restricted diet (Berry *et al.* 2004). Mean plasma galactose levels in CG patients on a galactose-restricted diet are on average 2.72 ± 0.70 μM (0.05 ± 0.01 mg/dL) compared to 0.52 ± 0.08 μM (0.01 ± 0.001 mg/dL) in non-galactosemic subjects on a lactose-free diet (Ning & Segal 2000); red blood cell gal-1P levels ranges from 1.1–3.5 mg/dL (0.04–0.13 mM) as compared to < 1 mg/dL (<0.038 mM) in non-galactosemic individuals (Berry *et al.* 1997).

### Complications of classic galactosemia

Beginning in infancy, individuals with CG are assessed periodically for cognitive development, neurological and motor functions, speech and language delays, psychosocial development, bone health, and cataracts (Welling *et al.* 2017). Further, gal-1P levels are checked regularly to determine dietary compliance, and adjustments are made for elevated levels. An observational study that included 33 adult participants (17 men and 16 women) with CG on a galactose-restricted diet found the following complications affecting participants: cataracts (21%), low bone density (24%), tremor (46%), ataxia (15%), dysarthria (24%), and apraxia of speech (9%) (Waisbren *et al.* 2012).

### Primary ovarian insufficiency in classic galactosemia

POI is one of the most common chronic complication associated with CG and affects 80–90% of female patients following a galactose-restricted diet (Fridovich-Keil *et al.* 2011). POI is defined as amenorrhea greater than 3 months in young adolescents and adult women who are less than 40 years of age with two follicle-stimulating hormone (FSH) levels in the menopausal range within a month of testing and decreased serum estradiol levels (Nelson 2009). POI can lead to infertility, hypoestrogenism, as well as mental distress (Nelson 2009).

The hypoestrogenic environment resulting from POI can result in poor bone health with an increased risk of fracture (Marino & Misra 2011, Batey *et al.* 2013), heightened risk of cardiovascular events (Roeters Van Lennep *et al.* 2016), and menopausal symptoms (Sullivan *et al.* 2016). Estrogen is the primary hormone that promotes secondary sexual characteristics that develop during puberty such as breasts, bone maturation, uterine growth, and the onset of menses. Many females with POI before pubertal onset will not develop these secondary sex characteristics. Hormone replacement therapy (HRT) with estrogen and progesterone is the mainstay treatment for patients with POI (see also 'Management,' subsequently).

### The onset of primary ovarian insufficiency in classic galactosemia

**Figure 1:**
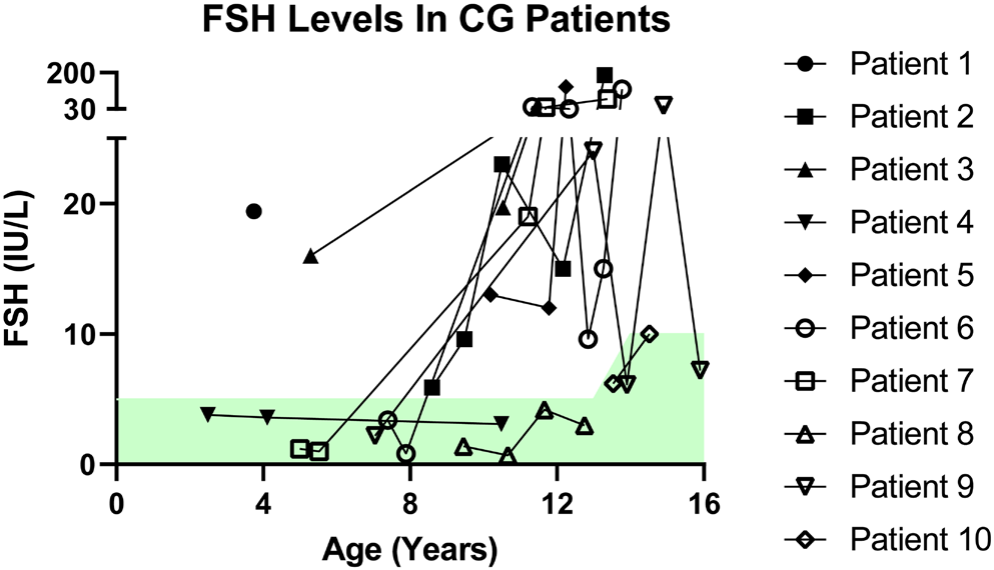
Longitudinal follicle-stimulating hormone (FSH) levels for female patients with classic galactosemia (CG) . FSH levels are provided for a cohort of child and adolescent CG patients. The green shaded region represents normal values. The timing of hormone replacement initiation was not available for this patient set; this may have influenced FSH levels in some patients.

Increasing evidence in CG POI suggests female infants have normal-appearing ovaries at birth that become damaged over time. A review of gonadal function in patients with CG by Rubio-Gozalbo and colleagues in 2010 included 23 female patients whose ovaries were evaluated either histologically, by imaging, or laparoscopy (Rubio-Gozalbo *et al.* 2010). Of the 23 patients, two were neonates and had morphologically normal ovaries with numerous oocytes (Levy *et al.* 1984, Levy 1996). The remaining patients, excluding one, were > of age and all but two had hypoplastic ovaries, and evidence of ovarian dysfunction with either fibrous ovarian tissue, significantly diminished primordial follicles, or absent growing follicles (Rubio-Gozalbo *et al.* 2010). There was a single patient that had normal-appearing ovaries at the age of seven, but repeat laparoscopy at age 17 revealed streak gonads (Kaufman *et al.* 1981). These findings are consistent with recent data from ovarian tissue cryopreservation (OTC) in prepubertal CG patients. Six prepubertal girls with CG underwent ovarian tissue cryopreservation for fertility preservation; five of the girls were less than five years of age and had normal ovarian morphology and number of follicles whereas the remaining girl was 11.7 years of age and had absent ovarian follicles (Mamsen *et al.* 2018). This further suggests that females with CG have normal oocyte numbers during infancy and early childhood with rapid loss over time. The trajectory of gonadotrophins to mirror menopausal levels throughout childhood into adolescence can further corroborate the biopsy/ultrasounds and suggest a progression of ovarian failure; although evidence of abnormal gonadotrophin levels have been seen as early as nine months in galactosemia (Kaufman *et al.* 1981, Levy *et al.* 1984). A retrospective chart review of 11 females with CG followed by our clinic was conducted. When available, information regarding genotype, the timing of menses/amenorrhea, the use of HRT and labs including gal-1P, FSH, anti-Müllerian hormone (AMH), luteinizing hormone (LH) and estradiol were recorded longitudinally for each patient. Our data revealed nine of 11 patients with evidence of POI including abnormal hormonal values. Of those nine patients, FSH levels before and after the age of 10 were available for six patients (summarized in Fig. 1). Four of the six had normal/borderline FSH levels prior to age 10 that sharply increased at age 10. The other two patients had elevated FSH levels prior to age 10. Six patients had data for AMH, with only two showing normal/high AMH levels before age 10 or when measured (Table 1).

**Table 1:** Patient data for genotype and timing of POI.

Patient no.	Genotype	Spontaneous menses	Time of amenorrhea	Hormone replacement therapy	Anti-Müllerian hormone (AMH)	Ovary images
1	Q188R/Q212X	Prepubertal	Prepubertal	Not indicated at this time	Low at 7 months and age 4	N/A
2	Q188R/Q188R	Yes	~16 years	Yes, periods stopped when hormones stopped	Low at age 28	N/A
3	S135L/IVS2-2G	Prepubertal	Prepubertal	Not indicated at this time	Low at ages 5–11	N/A
4	Q188R/R258C	Yes	None, regular periods	No	Normal range at age 16	N/A
5	Q188R/Q188R	Yes	~16 years	Yes, periods stopped when hormones stopped	Low at age 25	N/A
6	Q188R/unknown	Yes	21 years	Birth control as contraception	Low at ages 18–27	Yes; smaller than postmenopausal volume at age 27
7	Q188R/Q188R	Yes	None, regular periods	Yes, periods regular on birth control	Low at ages 18–22	N/A
8	Q188R/E203K	Yes	Unknown	No	High at ages 9–13	N/A
9	Q188R/Q188R	Yes	~16 years	Unknown	N/A	N/A
10	R201H/M336L	Yes	Unknown	Unknown	N/A	N/A
11	Q188R/Q188R	Yes	No	Birth control as a contraceptive	N/A	Yes; scan for second pregnancy at > 30 years of age showed no visible ovaries

While 32–55% of patients with CG do not achieve spontaneous menarche, a fraction that do can cycle regularly into their late 20s (van Erven *et al.* 2017, Frederick *et al.* 2018). Most cases of POI in CG are diagnosed in the pre-teen to teenage years due to ovarian insufficiency not being clinically evident until puberty (Fridovich-Keil *et al.* 2011). In a recent study that evaluated these parameters in the largest cohort of patients with CG to date, 68% of the 102 post-pubertal females with CG achieved spontaneous menarche. Spontaneous menarche occurred at an average age of 13.8 years (range 10–19), and HRT-assisted menarche was 14.2 years (range 11–18) (Frederick *et al.* 2018). If spontaneous menarche was achieved, only 50% of patients still cycled regularly after three years, 35% after five years, and fewer than 15% had regular menstrual cycles after 10 years. In the patients that did not receive HRT, approximately one-third of the 102 patients with CG had regular menstrual cycles in their late teens, and the number decreased to approximately 10% by their mid-20s (Frederick *et al.* 2018). In our small set of patients (Table 1), the majority did achieve spontaneous menarche, but only 50% were still cycling regularly after age 16 without the use of hormonal therapies (Table 1).

Due to the variability in the timing of POI onset among CG patients, and the lack of a reliable early screening tool for ovarian function/ovarian reserve, patients are often not officially diagnosed with POI until their peri-pubertal years when secondary sexual characteristics are absent (see also subsequent section). This makes counseling and prospective management of POI in CG difficult. Even so, some potential risk factors for the development of POI in CG patients have been identified. For example, there is a higher probability of POI and other long-term complications if the *GALT* mutation is Q188R/Q188R (Guerrero *et al.* 2000) or if the mean erythrocyte gal-1P is > 2.0 mg/dL on a galactose-restricted diet (Yuzyuk *et al.* 2018). The p.Q188R mutation is one of the most prevalent missense mutations in the *GALT* gene that leads to higher instability of the protein (Kumar *et al.* 2020). In addition to POI, homozygous Q188R mutations are also associated with cognitive impairment and verbal dyspraxia (Robertson *et al.* 2000, Antshel *et al.* 2004).

## Pathophysiology of primary ovarian insufficiency

Oocytes, which are located within ovarian follicles, are progressively lost throughout the female life cycle. The maximum number of oocytes (approximately 6–7 million) is before birth at around 20 weeks gestation, which is also when primordial follicles begin to form. At birth, there are approximately 1–2 million oocytes that drop to 300,000–400,000 at puberty, and only 400–500 are ovulated during a lifespan (Strauss III & Williams 2019). Oocytes are female germ cells that are capable of fertilizing with sperm to create embryos. Follicles consist of the cell types that envelop the oocyte, such as the granulosa cells and theca cells, and the oocyte itself. Intricate cellular communication between the oocyte and the surrounding granulosa cells determines the health and longevity of the ovary. LH and FSH are gonadotropins produced by the anterior pituitary that are important in regulating follicular growth, ovulation, and ovarian hormone production. Estrogen, which is a significant steroid hormone produced by granulosa cells of preovulatory follicles, is created via a two-cell process. LH stimulates theca cells to produce androgens, and FSH stimulates granulosa cells to aromatize the androgens into estrogen, which is then released into the circulation (Strauss III & Williams 2019). In women with POI, there is an accelerated loss of follicles by either destruction or atresia resulting in diminished ovarian function. Loss of ovarian function leads to long-term complications related to hypoestrogenism in addition to infertility. Multiple factors can cause POI including genetic variants, autoimmune disorders, metabolic causes, infectious causes, iatrogenic and toxic causes (Panay *et al.* 2020).

### Folliculogenesis and cellular signaling pathways

To delineate the potential pathophysiology of ovarian demise in CG, it is important to appreciate the normal lifespan of the ovary and follicle development. Folliculogenesis occurs in essentially four stages, beginning with the transition of a primordial follicle to the primary follicle, then to a secondary, antral, and finally, preovulatory follicle. Even in healthy ovaries, the vast majority of oocytes and follicles will not achieve ovulation but will die by atresia (Liu *et al.* 2006, Adhikari & Liu 2009). *In utero*, primordial follicles undergo meiosis and remain arrested at prophase I of the cell cycle. At around 24 weeks gestation, the first wave of primordial follicles become activated and grow into primary follicles, and after birth, the majority grow and are arrested at the pre-antral stage. At puberty, gonadotrophin production begins and selected follicles can progress to the antral stage with a select number maturing to ovulate an oocyte (Ford *et al.* 2020). The growth of primordial follicles to ultimately ovulatory follicles is a continuous process throughout a woman’s reproductive life, and the number of primordial follicles is considered the ovarian reserve; thus, the appropriate timing is integral in preserving ovarian function until anticipated menopause.

Various animal models of POI and cultured human ovaries have elucidated different signaling pathways involved in folliculogenesis at different stages of development; of note for this review are the phosphatidylinositol 3-kinase/AKT signaling pathway (PI3K/AKT), and the unfolded protein response (UPR), specifically EIF2A phosphorylation (Ernst *et al.* 2017). Many other well-known signaling pathways, including nuclear receptor signaling (Komatsu & Masubuchi 2017, Chakravarthi *et al.* 2020), TGFB (Yoon *et al.* 2013, Chang *et al.* 2016), Hippo (Hsueh & Kawamura 2020), and the NFKB pathway (Wright *et al.* 2020) have all been shown to regulate normal folliculogenesis. Here, we will limit our discussion to signaling pathways that have been investigated in the context of ovarian dysregulation in the CG mouse model.

Tightly regulated control of PI3K/AKT signaling is paramount for proper initiation of follicle growth and primordial follicle survival (Liu *et al.* 2006). Loss of regulators of PI3K/AKT signaling such as *Pten*, *Foxo3a*, *Pdk1*, and *Tsc 1/2* in mouse oocytes and granulosa cells can lead to abnormal activation of primordial follicles, and thus, POI with depletion of the ovarian reserve (John *et al.* 2008, Jagarlamudi *et al.* 2009, Reddy *et al.* 2009, Adhikari *et al.* 2010). Disruption of upstream regulators of PI3K/AKT such as semaphorin 6C (SEMA6C) and cell division cycle 42 (CDC42) can also influence follicle activation through PI3K/AKT signaling (Yan *et al.* 2018, Zhou *et al.* 2018). The phosphorylation of AKT is central in PI3K/AKT signaling and controls growth in the cell. In mice with *Pten* deleted in their oocytes, increased pAKT was associated in a stage-specific manner with the mass activation of follicles (Jagarlamudi *et al.* 2009). Additionally, stimulation of AKT phosphorylation and inhibition of PTEN in cortical fragments of ovaries from human patients receiving chemotherapy were able to activate primordial follicles and produced two live human births (Kawamura *et al.* 2013, Suzuki *et al.* 2015). Changes in PI3K/AKT signaling are also associated with a high-fat diet; Nteeba and coworkers saw increased levels of mRNA for AKT and Kit ligand indicating enhanced PI3K/AKT signaling in older mice after a high-fat diet (Nteeba *et al.* 2013). While PI3K/AKT signaling is involved in the activation and growth of follicles, the exact mechanism and timing of activity in this pathway are poorly understood.

The UPR is a time-dependent, paradoxical survival or death response in the cell in response to ER stress that culminates in either the cell restoring homeostasis or dying *via* apoptosis and/or autophagy (Donnelly *et al.* 2013). Improper folding of proteins (a surge of protein synthesis), abnormalities in glycosylation, increased oxidative stress, and perturbations of Ca^2+^ homeostasis are all activators of ER stress and the UPR (Oslowski & Urano 2011). Harada and coworkers found evidence of increased UPR molecular markers in the granulosa cells of late secondary follicles in mice suggesting a normal physiological role of UPR in meeting the needs of increased protein synthesis as the follicle nears maturation (Harada *et al.* 2015). True to the roles of the UPR as both pro-survival and death promoter, in goat ovaries, ER stress marker protein BiP was present in both atretic and non-atretic granulosa cells but increased in the granulosa cells of follicles destined for atresia; early atretic follicles also showed increased BiP (Lin *et al.* 2012). In contrast, the apoptosis marker CHOP was only present in granulosa cells of atretic follicles (Lin *et al.* 2012). *In vitro* and *in vivo* models of obesity-induced POI exhibited increased ER stress markers and saw less viable oocytes ovulated; administration of a UPR modulator (Salubrinal) reversed these poor outcomes (Wu *et al.* 2010, 2012, 2015). As the UPR is a phenomenon in both follicle growth and atresia, any disruption of the UPR likely will be detrimental to the oocyte, follicles, and ovarian health (Lin *et al.* 2012, Babayev *et al.* 2016, Xiong *et al.* 2017, Takehara *et al.* 2020).

One key regulator of the UPR is the phosphorylation of EIF2A. In times of cellular stress, the phosphorylation of EIF2A serves to slow down protein translation in the cell to restore homeostasis (Donnelly *et al.* 2013). Beyond the UPR, EIF2A is an important component of normal protein translation, specifically to the 43S pre-initiation complex which acts with the EIF4F complex to initiate synthesis of new polypeptides during cellular growth (Colegrove-Otero *et al.* 2005). The phosphorylation of EIF2A by various kinases blocks the initiation of protein translation by inhibiting EIF2B, the nucleotide exchange factor involved in the 43S pre-initiation complex (Donnelly *et al.* 2013). In the oocyte, phosphorylated EIF2A appears to be involved in regulating meiosis and keeping follicles dormant until their predestined activation (Le Bouffant *et al.* 2008, Alves *et al.* 2009). As keeping the lifespan of follicles dormant is critical for ovarian longevity, EIF2A regulation is likely a key player in developing POI. In humans, mutations in *EIF4ENIF1*,an important transport protein for components crucial to the EIF4F complex, result in POI (Kasippillai *et al.* 2013, Zhao *et al.* 2019). Mutations in *EIF2B* are also associated with POI with concurrent neurological complications, providing more evidence that protein initiation factors are involved in maintaining ovarian health (Fogli *et al.* 2004).

### Human and animal model examples

POI is associated with a variety of different conditions such as X chromosome abnormalities (Turner syndrome, *FMR1*), autosomal recessive disorders (CG, mitochondrial diseases), autoimmune diseases (Addison’s disease), specific single-gene defects (*EIF4ENIF1*, E*IF2B*, *FOXL2*, *GDF9*, *FOXO3*, *FSHR*,etc.), and iatrogenic causes (chemotherapy) (Rossetti *et al.* 2017). Also, metabolic dysfunction such as obesity is associated with problems in reproduction, including POI (Hohos & Skaznik-Wikiel 2017). While the etiology of POI in all causes is largely unknown, the pathophysiology of POI can be divided into two major categories: developmental problems with primordial follicles resulting in atresia before growth or by follicle depletion through accelerated growth and atresia (Panay *et al.* 2020).

We can look to other examples of POI caused by damaging environments such as autoimmune, iatrogenic, metabolic syndrome (obesity/high-fat diet), and models of galactose-intoxicated animals to speculate how the follicle damage is caused by abnormal metabolites in CG. Various studies exploring autoimmune causes of POI show that the oocyte, granulosa cells, zona pellucida, steroid hormone-producing cells in developing follicles, and the pituitary–hypothalamic axis can all be a target of harmful antibodies (Forges *et al.* 2004, Altuntas *et al.* 2006, Sharif *et al.* 2019). Induced oophoritis in animal models reveals damage mainly to growing and developing follicles (Domniz & Meirow 2019). Targeted antibody attack of one negative regulator of FSH led to increased FSH and follicle depletion due to increased follicle activation early in life (Altuntas *et al.* 2006). Chemotherapy agent cyclophosphamide appears to cause POI by damaging DNA, specifically in the primordial oocyte and granulosa cells of developing follicles. The damage to the primordial oocyte leads to a brief moment of growth in the oocyte but then to apoptosis without differentiation to a primary follicle at day one after administration of cyclophosphamide (Luan *et al.* 2019). There are conflicting interpretations of whether chemotherapy causes primordial follicle death and no increased activation of primordial follicles (Luan *et al.* 2019) or POI due to increased activation (Kalich-Philosoph *et al.* 2013); regardless, it is clear that primordial follicle damage contributes to developing POI. Researchers also saw changes in AMH levels after the administration of cyclophosphamide in mice. Three days after administration, AMH fell dramatically, similar to what is seen in humans with follicle damage, and then steadily increased to control levels seven days post-treatment, presumably due to improvement in follicle health after the initial insult (Luan *et al.* 2019).

Obesity, specifically a high-fat diet, can lead to reproductive dysfunction such as follicular and oocyte damage, lower responses to *in vitro* fertilization (IVF), and problems in the hypothalamic–pituitary–ovarian (HPO) axis likely due to high levels of insulin (Luke *et al.* 2011, Broughton & Moley 2017, Hohos and Skaznik-Wikiel 2017). Limited mechanistic studies of obesity/high-fat diet and the ovary are available in humans beyond association data; however, women fed a high-fat diet for two to four menstrual cycles experienced characteristics of POI such as cycle disruption, a shorter time between menses, increased FSH, and decreased estradiol (Hill *et al.* 1984, Reichman *et al.* 1992). Machtinger and colleagues assessed the quality of oocytes from obese women that failed fertilization during IVF and found disruptions in spindle and chromosome alignment (Machtinger *et al.* 2012). Additionally, women with POI are at a greater risk of developing type 2 diabetes, suggesting a metabolic relationship between the two disorders (Anagnostis *et al.* 2019). Animal studies have shed more light on the potential mechanism causing the disruptions related to obesity such as oxidative stress, mitochondrial dysfunction, HPO axis disruption, and problems in PI3K/AKT signaling (Wu *et al.* 2010, 2012, Nteeba *et al.* 2013). In mouse models of obesity, high-fat diet or genetically obese mice have ovarian aging and evidence of POI. Female mice fed high-fat diets had fewer primordial follicles, increased developing follicles, and more corpus luteum than normal caloric or restricted caloric mice (Wang *et al.* 2014).

Galactose-intoxicated rat models can give some clues to the toxic effects of galactose on the ovary (Rostami Dovom *et al.* 2019). Early prenatal administration (before 15 days post-conception) of 35% of calories from galactose to mother rats resulted in decreased primordial follicles, increased apoptosis, and disruption in germ cell formation in the pups, suggesting follicle damage in utero (Bandyopadhyay *et al.* 2003*a*,*b*). Other studies found that prenatal and postnatal administration of high galactose (>30%) caused increased FSH and LH and decreased estradiol, similar to human gonadotrophic presentations in POI (Chen *et al.* 1981). In these studies, the primary metabolite measured was gal-1P; however, the proximal mechanism that contributes to the follicle demise in these models is unknown (Rostami Dovom *et al.* 2019). Postnatal exposure to high galactose (40%) after weaning increased galactitol concentrations in the ovary and resulted in impaired folliculogenesis; administration of an aldose reductase inhibitor ameliorated the effects of galactitol toxicity suggesting that galactitol and the oxidative stress it creates when it is reduced are potential contributors to ovarian damage in POI in experimental hypergalactosemia. In this study, gal-1P was not measured.

### Pathophysiology of primary ovarian insufficiency in galactosemia

The aforementioned models of POI and human examples give clues about what types of insults can cause POI. However, while informative to the development of POI, these models are not specific to CG, as there are presumed normal functions of the GALT enzyme. Indistinct prenatal insults are possible in CG female ovaries due to endogenous production of galactose; however, this has only been examined in galactose-intoxicated rat models of POI and not measured in human fetuses (Bandyopadhyay *et al.* 2003*b*). While the exact mechanism of ovarian failure is not known, overwhelming evidence suggests that excess gal-1P is the main culprit in CG POI (Guerrero *et al.* 2000, Bandyopadhyay *et al.* 2003*a*, Tang *et al.* 2014, Thakur *et al.* 2017, Vanoevelen *et al.* 2018, Yuzyuk *et al.* 2018, Balakrishnan *et al.* 2019). Females heterozygous for pathogenic variants in *GALT* and with other types of galactosemia, such as GALK1 deficiency, do not have chronically elevated levels of gal-1P, have a normal ovarian reserve, and do not undergo menopause at a premature age (Knauff *et al.* 2007, Badik *et al.* 2011, Rubio-Gozalbo *et al.* 2021). A summary of studies that evaluate the 'toxic' action of specific galactose metabolites is presented in Table 2.

**Table 2:** Evidence for galactosemia metabolite toxic action.

Species/Aberrant metabolites	Consequences	Mechanism	References
Humans			
Gal-1P	Increased risk of long-term complications in humans	Unknown	Yuzyuk *et al.* (2018)
Gal-1P	Increased risk of POI in humans	Unknown	Guerrero *et al.* (2000)
Gal-1P	Best predictor for verbal dyspraxia	Unknown	Webb *et al.* (2003)
Gal-1P	Inhibits growth in fibroblasts derived from galactosemic patients	UDP-hexose deficiency	Lai *et al.* (2003)
Gal-1P	Increased stress in GALT negative human-derived fibroblasts	Accumulation of unfolded proteins, altered calcium homeostasis, and ER stress	Slepak *et al.* (2007)
Mouse			
D-galactose Gal-1P	Damages MII mouse oocytes and hinders embryo development	Increased ROS and disruption of spindle structure and chromosomal alignment	Thakur *et al.* (2017)
Galactose	Decreased oocyte number in offspring	Unknown	Chen *et al.* (1981)
Gal-1P	Subfertility, follicular dysfunction, and growth restriction in GALT-deficient mouse models		Tang *et al.* (2014)
Gal-1P	Reduced growth of mutant mouse fibroblasts	Increased ER stress in mouse fibroblasts via regulation of PI3K/Akt signaling	Balakrishnan *et al.* (2016)
Gal-1P	Ovarian dysfunction in galactosemia mouse model	Increased ER stress via regulation of PI3K/Akt signaling	Balakrishnan *et al.* (2019)
Gal-1P	Increased ataxia in galactosemia mouse model	Stress-related cellular damage via regulation of PI3K/Akt signaling	Chen *et al.* (2017)
Galactose, galactitol, Gal-1P	Follicular atresia	Unknown	Bandyopadhyay *et al.* (2003*a*)
Galactose	Adverse germ cell migration with initial low pool of germ cells	Unknown	Bandyopadhyay *et al.* (2003*b*)
Galactitol	Cataracts	Unknown	Ai *et al.* (2000)
Yeast			
Gal-1P	Growth arrest in yeast models	Increased environmental stress	Slepak *et al.* (2005)
Gal-1P	Growth arrest in galactosemia yeast models	Unknown	Ross *et al.* (2004)
Gal-1P	Decreased growth rate in yeast	Decrease in intracellular phosphate levels	Machado *et al.* (2017)
Gal-1P	Growth arrest of GALT negative yeast		Mumma *et al.* (2008)
Gal-1P	Decreased growth rates in galactosemic yeast model	Inhibition of phosphoglucomutase	de Jongh *et al.* (2008)
Zebrafish			
Gal-1P	Reduction in motor function and fertility potential	unknown	Vanoevelen *et al.* (2018)

The *GALT* knockout zebrafish and mouse model have the POI phenotype similar to CG humans (Tang *et al.* 2014, Vanoevelen *et al.* 2018). Zebrafish with *galt*-knockout show increased gal-1P, decreased number of eggs after fertilization, and increased galk1 and gale activity in the ovary (Vanoevelen *et al.* 2018). This model showed aberrant glycosylation and disturbed sialyation in the ovary (Haskovic *et al.* 2020). Previously, our lab developed a new homozygous *GalT*-gene trapped mouse model to study the pathophysiology of CG on a multicellular organism (Tang *et al.* 2014). Galactose sensitivity, reduced fertility in females, hypogalactosylation of IgG, growth restriction, and motor impairment are seen in the GalT-deficient mice in both sexes, similar to humans with CG (Balakrishnan *et al.* 2016, 2017, Chen *et al.* 2017).

In our mouse model, we see subfertility, reduced follicles, increased corpus luteum, increased ER/UPR stress markers, and decreased PI3K/AKT signaling in two-month-old ovaries compared to WT ovaries (Balakrishnan *et al.* 2017, 2019). While there were significantly fewer primordial follicles at two months of age, follicle counts at postnatal day eight (unpublished data) showed no difference in primordial follicles when compared to WT mice, suggesting that follicle loss occurs progressively between birth and sexual maturity in a mouse, similar to case reports in females with CG (Kaufman *et al.* 1981, Balakrishnan *et al.* 2019). Recently, we used Salubrinal to modulate the UPR signaling in our GALT-deficient mouse model (Balakrishnan *et al.* 2019). Salubrinal inhibits the dephosphorylation of EIF2A, keeping protein translation reduced in the cell. The administration of Salubrinal at five weeks of life improved the fertility of GALT-deficient female mice by normalizing estrus cycles and increasing the number of pups born per litter (Balakrishnan *et al.* 2019). Histologic analysis of ovaries from Salubrinal-treated mice showed an increased number of primordial follicles when compared to untreated GALT-deficient mice (Balakrishnan *et al.* 2019).

It is reasonable to assume that the administration of Salubrinal, which allows for increased pEIF2A, may help to keep primordial follicles quiescent, and thus, preserve ovarian function. However, it is unknown if ovarian failure in CG is due to premature activation of primordial follicles or follicle atresia before activation. Preliminary data from our lab comparing one-month-old ovaries showed an increased ratio of primary to primordial follicles in GALT-deficient mice suggesting premature follicle activation and thus, depletion before sexual maturity (unpublished data). Immunofluorescent histological studies show evidence of increased ER stress marker BiP at two weeks of life in the granulosa cells of follicles in GALT-deficient ovaries (unpublished data).

Our lab found decreased markers of PI3K/AKT signaling in GALT-deficient fibroblasts and whole ovaries at two months of age compared to age-matched WT mice (Balakrishnan *et al.* 2016, 2017). The ovarian histology revealed a significant decrease in ovarian follicles of all stages and increased corpus luteum tissue (Balakrishnan *et al.* 2017). Interestingly, after administration of Salubrinal to GALT-deficient mice, molecular markers of PI3K/AKT signaling were improved in the whole ovary as well as an increased number of follicles (Balakrishnan *et al.* 2019). The relationship between the UPR and PI3K/AKT signaling is not well understood, but it appears that there is a communication between the phosphorylation of EIF2A and the phosphorylation of AKT (Qin *et al.* 2010, Tian *et al.* 2011). As PI3K/AKT signaling is tightly regulated in follicle activation, we speculate that by keeping eIF2ɑ phosphorylated, proper signaling is restored in the primordial follicle, allowing for follicle quiescence. Differences in PI3K/AKT signaling at earlier ages have not been explored in our mouse model of CG or the specific cell types involved. If there is evidence of early, mass primordial follicle activation in GALT-deficient mice, immunofluorescent studies may reveal increased PI3K/AKT signaling in oocytes/granulosa cells at earlier time points in development (Jagarlamudi *et al.* 2009). Modulators of PI3K/AKT signaling could also be used as a potential treatment if indicated, once the pathophysiology of POI in galactosemia is elucidated.

## Clinical considerations

### Evaluation of POI in galactosemia

The clinical presentation for CG-induced POI can manifest as delayed or absent puberty, primary or secondary amenorrhea, oligomenorrhea, or infertility (Fridovich-Keil *et al.* 2011, Thakur *et al.* 2018). In 2017, members of The Galactosemia Network (GalNet) developed evidence-based guidelines for the diagnosis, treatment, and follow-up of CG including specific recommendations related to endocrinology and fertility (Welling *et al.* 2017). The guidelines recommend that all females with CG be screened for POI by the age of 12 with absent secondary sexual characteristics or if they reach age 14 with irregular menses, and screening should include serum FSH and estradiol measurements. Additionally, the guidelines also recommend that females with CG that have undergone puberty with regular ongoing menstrual periods be monitored annually for signs or symptoms of POI and FSH measurement if symptoms arise (Welling *et al.* 2017). We provide a summary of guidelines for clinical management specific to CG and more broadly to POI in Table 3.

**Table 3:** Clinical management of galactosemia and POI.

Age	Guidelines	Unknowns	Considerations	Supporting references
Newborn	Screening for galactosemiaImplementing dietary management			
1–11 years	Monitor Gal-1P levels regularly to ensure dietary compliance	Utility of monitoring ovarian reserve to evaluate for imminent POIAge to consider fertility preservationThe role ER stress blockers play in maintaining fertility potential	Draw FSH and AMH annuallyConsider fertility preservation options (OTC vs oocyte cryopreservation) under research protocol as follicular numbers appear to be normal in younger patients	(Mamsen *et al.* 2018, Frederick *et al.* 2018, Yuzyuk *et al.* 2018, Azem *et al.* 2020, Poirot *et al.* 2019, Segers *et al.* 2020, Sanders *et al.* 2009)
12–14 years	Monitor secondary sexual characteristics and menarcheScreen for POI (FSH, E2) if no secondary sexual characteristics by age 12 or irregular menses by age 14Initiate HRT if POI diagnosed	Is there a role for routine fertility preservation in this age groupThe role ER stress blockers play in maintaining fertility potential	Recommend adding AMH to screening protocol for POI as it appears to be predictive of spontaneous menarcheConsider fertility preservation in patients with evidence of adequate ovarian reserve	(Mamsen *et al.* 2018, Frederick *et al.* 2018, Cobo *et al.* 2016, Poirot *et al.* 2019, Segers *et al.* 2020)
14+ years	Monitor annually for menstrual changes and symptoms of POI in women who underwent puberty. Screen for POI with concerns (FSH)Initiate HRT for newly diagnosed POICounsel on spontaneous conception and implement contraception if neededRefer to REI for fertility considerationsScreen for emotional well-being	Is there a role for routine fertility preservation for patients with adequate ovarian reserveThe role ER stress blockers play in maintaining fertility potential in patients who have completed puberty	Include AMH as routine screening for POIConsider fertility preservation (OTC vs oocyte cryopreservation) in patients with evidence of adequate ovarian reserve	(Mamsen *et al.* 2018, Frederick *et al.* 2018, Yuzyuk *et al.* 2018, Cobo *et al.* 2016, Poirot *et al.* 2019, Segers *et al.* 2020, Sanders *et al.* 2009)

Alternative methods for screening for POI have been evaluated including measuring AMH levels. AMH is produced by granulosa cells of pre-antral and antral follicles, and its use has become more widespread in measuring ovarian reserve (Hagen *et al.* 2010, Hansen *et al.* 2011). While the guidelines currently recommend against using AMH as a screening tool for POI in this patient population (Welling *et al.* 2017), there have been several studies that have evaluated AMH levels in CG-induced POI (Spencer *et al.* 2013, Frederick *et al.* 2018). Studies show that patients with CG have significantly lower AMH values than controls, and AMH values can be assessed before menarche) or after initiation of HRT (Sanders *et al.* 2009, Spencer *et al.* 2013) supporting its potential utility for screening. More recently, Frederick and colleagues (Frederick *et al.* 2018) measured both AMH and FSH in patients with CG and tested for possible association(s) with spontaneous menarche. They found that a detectable AMH level (≥0.04 ng/mL) was highly predictive of spontaneous menarche. FSH values, unlike AMH, did not associate with spontaneous menarche. AMH values are also useful in screening for POI in patients with Turner syndrome (Hagen *et al.* 2010, Lunding *et al.* 2015). In prepubertal patients, an AMH less than 0.56 ng/mL predicted the absence of puberty in Turner syndrome girls (Lunding *et al.* 2015). With recent advances in AMH assay sensitivity as well as evidence of its utility in predicting menarche, we recommend routinely checking AMH levels in prepubertal patients with CG and those that have not been diagnosed with POI.

### Management of POI in galactosemia

In CG patients with primary amenorrhea and absent puberty, the GalNet guidelines recommend starting with low-dose estradiol and increasing the dosage in a step-wise fashion to mimic physiological ovarian hormone function, with progesterone supplementation at a later time to induce withdrawal bleeding for endometrial protection (Welling *et al.* 2017). The exact timing of when to start HRT for young girls with CG and POI has not been well established but is generally started once the patient is at a peri-pubertal age, and the diagnosis of POI has been made.

According to the ESHRE guidelines on POI in the general population, low-dose estradiol should be started between ages 12–13 if no secondary sexual characteristics are present, and FSH is elevated. Estradiol should be gradually increased every 6–12 months for 2–3 years until an adult dosage is reached, and cyclic progesterone should be started after two years of estradiol or with the onset of breakthrough bleeding (Webber *et al.* 2016). There are many different routes of estrogen replacement including oral, transdermal, and transvaginal (Sullivan *et al.* 2016) with no form showing superiority in regards to improvement in symptoms (Webber *et al.* 2017); however, transdermal and transvaginal routes show decreased occurrence of venous-thromboembolism (VTE) in postmenopausal patients, but this has not been shown in younger populations (Webber *et al.* 2017, Vinogradova *et al.* 2019). However, given the duration of therapy in this population and the paucity of long-term longitudinal studies in pre-menopausal women with POI, mitigating VTE risk with transdermal and transvaginal administration may be prudent (Canonico *et al.* 2014).

Progesterone supplementation is traditionally given orally in either a micronized progesterone form or as a synthetic progestogen such as medroxyprogesterone acetate in a cyclical manner to induce regular menstrual cycles; it also can be given continuously in patients that do not desire a menstrual cycle. Alternative forms of progesterone are available such as transdermal, transvaginal, and intrauterine including the levonorgestrel intrauterine device (LNG-IUD) which supplies endometrial protection while also allowing for contraception (Webber *et al.* 2017). Some patients may prefer combined oral contraceptive pills (COCPs) as an alternative to traditional HRT as this is often more socially acceptable in their age group. However, studies have suggested that COCPs are inferior to HRT in maintaining bone health, but sample sizes have been small (Crofton *et al.* 2010, Cartwright *et al.* 2016). Patients who elect COCP use for HRT long-term should also be advised of the lack of improvement in associated genitourinary symptoms with this formulation compared to 17-beta estradiol as well as the lack of potential cardiovascular protection. Patients with CG should be counseled to take the COCP continuously and skip the placebo pills which would render them without estrogen or progesterone for 25% of the time (Webber *et al.* 2017). Finally, individuals should be counseled that HRT is not a form of contraception, and if pregnancy is not desired, a form of contraception should be recommended.

Psychosocial parameters including social well-being and social functioning are lower in patients with CG compared to the general population (Hoffmann *et al.* 2012). These patients are also prone to anxiety and depression with one observational study showing 67% of CG subjects reporting anxiety and 39% reporting depression (Waisbren *et al.* 2012). Also, decreased emotional well-being, anxiety, and depression are also significant factors associated with POI (Nelson 2009). Young women diagnosed with 'early menopause' may feel stigmatized and often devasted at the diagnosis especially when understanding the current incurable nature of the disease, related infertility, and being different than their peers (Sterling & Nelson 2011). A cross-sectional and case-control study by Davis and colleagues comparing spontaneous POI in women with 46,XX with a mean age of 32.4 years to age-matched controls found that those with POI had increased anxiety, depression, and negative affect as well as decreased overall well-being (Davis *et al.* 2010). Patients also perceive a lack of psychosocial support after receiving the diagnosis of POI, and few receive appropriate follow-up from social workers or a psychologist (Kanj *et al.* 2018). Appropriate counseling regarding the diagnosis as well as psychological support is extremely important in this patient population, and perhaps a multidisciplinary team approach is the best management method.

## Fertility management

Infertility is a major long-term effect in patients with POI, although there are reports of spontaneous pregnancy in this population. The ovarian function in these women is limited, but erratic and unpredictable ovulation can still occur. In the broader POI population, 5–10% spontaneously conceive (van Kasteren & Schoemaker 1999). As mentioned previously, patients with CG continue to have ovarian compromise despite being on a galactose-restricted diet and many eventually experience POI. Yet, recent data has suggested fertility rates are higher in those patients with CG-induced POI compared to other causes of POI. In a study of 22 CG women, nine attempted to conceive spontaneously and four were successful (44% pregnancy rate) (Gubbels *et al.* 2008). Similar findings were detected in a recent observational study that evaluated 85 women with CG. In those patients, 21 actively attempted to conceive and nine achieved pregnancy (42.9% pregnancy rate) (van Erven *et al.* 2017). Even in CG patients with undetectable AMH levels and menopausal FSH levels, pregnancy can be achieved (Gubbels *et al.* 2009) (see as an example, Table 1). Ovarian reserve markers like FSH, AMH, and antral follicle count can help predict ovarian reserve and fertility potential but are not definitive in determining which patients with POI will and will not conceive. It is important to counsel patients appropriately on the chance of spontaneous conception and despite the diagnosis of POI, and for patients that do not desire pregnancy, contraception is recommended (Welling *et al.* 2017).

For those patients that do not achieve spontaneous conception and desire to conceive, referral to a reproductive endocrinologist to discuss fertility options such as oocyte and embryo donation should occur with adoption also being an alternative option (Baker 2011). Patients with POI are generally not ideal candidates to undergo IVF using autologous oocytes due to the extremely low chances of success secondary to depleted follicular reserve; while oocyte donation from a donor < 35 years of age would yield a live birth rate of approximately 44% (Hogan *et al.* 2019). Maintaining uterine health with estradiol and progesterone replacement therapy is thus important in helping these women achieve their family building goals.

### Fertility preservation

Fertility preservation options are studied more in patients undergoing gonadotoxic therapy such as chemotherapy for cancer treatment that would render them at high risk for ovarian insufficiency and infertility, but it should also be offered to people with more benign conditions that put them at high risk for ovarian insufficiency (Donnez & Dolmans 2017). Historically, the two standard methods for fertility preservation have been oocyte cryopreservation and embryo cryopreservation which have been utilized for over 30 years. With either method, the patient receives exogenous gonadotropins for ovarian stimulation, and the resulting growing ovarian follicles are then aspirated to retrieve the oocytes. The oocytes can either be frozen to be used for embryo creation at a later date, or the fresh oocytes can be fertilized with partner or donor sperm after the retrieval to create embryos. The resulting embryos are grown to the blastocyst stage and are frozen to be thawed and transferred at a later date. In patients with a good prognosis (i.e. 35 years of age or younger), 10 oocytes cryopreserved yield a cumulative live birth rate of 60.5%, and 15 oocytes yield a cumulative live birth rate of 85.2.% (Cobo *et al.* 2016). Unfortunately, due to the rapid depletion of the ovarian reserve pool often before puberty in patients with CG, oocyte and embryo cryopreservation are generally not a practical option for fertility preservation in this population. To date, there are no studies evaluating the utility of oocyte cryopreservation in post-puberal patients with CG that have retained ovarian function. Due to the immaturity of the HPO axis in prepubertal girls, exogenous stimulation with gonadotropins for oocyte cryopreservation has historically not been viewed as a viable option, but a recent publication has challenged this approach. Azem and colleagues report the case of a seven-year-old female with Turner syndrome mosaicism that underwent controlled ovarian hyperstimulation with six mature oocytes cryopreserved (Azem *et al.* 2020). Successful oocyte cryopreservation in a prepubertal patient at high risk for developing POI is an exciting potential option for patients with CG but will require further studies. Considerations include patient maturity to undergo stimulation at this age as well as utilization of transabdominal ultrasound for follicular monitoring with transvaginal retrieval under anesthesia.

OTC, which requires surgical removal of ovarian tissue cortex that is subsequently frozen and re-implanted at a later date, is becoming a more widespread and promising option that has also resulted in > 130 live births (Donnez & Dolmans 2017, Gellert *et al.* 2018). Recently, ASRM lifted the experimental label for OTC that can now be offered as a standard of care (Medicine ECotASfR 2018). Traditionally in prepubertal females, OTC has been seen as the only option for fertility preservation which was generally completed under a research protocol (Medicine ECotASfR 2018, Nahata *et al.* 2020). Currently, there is limited evidence regarding the safety and effectiveness of OTC in prepubertal patients with only one live birth as a result of OTC from a prepubertal patient (Matthews *et al.* 2018). However, there have been several reports of successful induction of puberty with tissue harvested from prepubertal girls, cryopreserved, and transplanted in early adolescence supporting the efficacy of prepubertal OTC (Ernst *et al.* 2013, Poirot *et al.* 2019). Regarding CG, there are no data concerning OTC in postpubertal patients, but recent data show a potential benefit for OTC in pre-pubertal patients with CG. Six girls with galactosemia below the age of 12 underwent OTC for fertility preservation. Normal ovarian follicular density and morphology were observed in five of the girls (all ages 5 or younger) compared to controls that underwent OTC before receiving gonadotoxic therapy. The remaining girl with CG that underwent OTC was 11.7 years, and there were no ovarian follicles detected (Mamsen *et al.* 2018).

*In vitro* maturation (IVM) is the process of maturing cumulus-oocyte complexes (COCs) to the metaphase II stage in a culture which can then be used for embryo creation or oocyte cryopreservation. Improvements in lab techniques have resulted in significant improvement in IVM success. A recent retrospective study included 77 patients who underwent OTC with the maturation of COCs obtained during ovarian tissue processing. IVM rate of immature oocytes to the metaphase II stage was 39%. Of the 77 patients, 64 had oocytes cryopreserved, and the live birth rate per patient that underwent ovarian tissue oocyte IVM followed by fertilization and embryo transfer was 43% (Segers *et al.* 2020).

Overall, there is a lack of evidence of the utility of fertility preservation in patients with non-oncologic causes of POI specifically in the prepubertal population. In particular, in patients with CG-induced POI, there is concern that the ovarian damage might have already occurred yielding OTC futile, and this process of removing ovarian tissue will further decrease the ovarian reserve in a population that is at high risk for POI with spontaneous conception still being possible (van Erven *et al.* 2013). Partial oophorectomy for OTC may be considered to allow retention of tissue in the event of spontaneous ovulation and conception. Currently, the guidelines do not recommend for patients with CG to undergo routine fertility preservation techniques, and these procedures should only be offered under research studies at young prepubertal ages (van Erven *et al.* 2013, Welling *et al.* 2017). Given the recent studies, albeit limited numbers, it appears young prepubertal girls less than five years of age with CG have normal ovarian follicular numbers and morphology. These findings coupled with the advancements in OTC and IVM show this as a promising method for fertility preservation in this patient population. Further studies need to be completed, but it is reasonable to discuss this option with patients and refer them to specialized centers as needed.

## Limitations and future directions

There are limitations to our review, including the lack of fertility preservation data on postpubertal patients with CG. Due to the high prevalence of early-onset POI, there are no studies evaluating fertility preservation in this age group. The considerations for fertility preservation in this population are extrapolated from the successes in similar patients with adequate ovarian reserve, which may influence clinical recommendations. The ER stress modulator Salubrinal is for research only and not safe for human use. Additionally, no data exists using ER stress modulators in human models of POI, making this target difficult to translate to a clinical setting. Finding FDA-approved drugs or nutraceuticals with similar mechanisms of action could be considered. Finally, the review of human and animal models of POI presented here are not specific to GALT deficiency, and the mechanisms that cause POI in the other models may not apply to CG. The mechanism of follicle demise may be very specific to the metabolites and cellular metabolism present in CG.

After over 50 years of research, the true pathophysiology of galactosemia remains unclear (Rubio-Gozalbo *et al.* 2019). No treatments for POI in galactosemia or the general population are used except for HRT, which only counters the effects of early menopause and does not prevent ovarian failure (Hadji 2008). Currently, gene/mRNA therapies and GALK1 inhibitors are being developed to treat GALT deficiency (Lai *et al.* 2014, Hu *et al.* 2019, Balakrishnan *et al.* 2020). However, it may be several years before this is available to human patients. Inhibitors of aldose reductase (AR), the enzyme responsible for converting excess galactose to galactitol, are also being explored as a potential treatment for galactosemia. AR inhibitors have the potential to be effective in protecting the ovary in particular from oxidative stress, which has been studied in a galactose-intoxicated rat model, but its relevance in patients with a galactose-restricted diet remains to be seen (Meyer *et al.* 1992). While Salubrinal showed improved fertility and increased primordial follicles in GALT-deficient mice (Kobayashi *et al*. 1999), its safety in humans is not well studied. At this time, compounds with a similar mechanism of action to Salubrinal and specific dietary supplements that are either FDA-approved or generally recognized as safe (GRAS) in humans are being tested as a treatment for POI in galactosemia. If a potential therapy is found, the timing of treatment for females with galactosemia remains to be determined.

## Declaration of interest

The authors declare that there is no conflict of interest that could be perceived as prejudicing the impartiality of this review.

## Funding

This work was supported the National Institutes of Health (grant number R01HD089933, PI: Lai), and by University of Colorado School of Medicine, Department of Obstetrics and Gynecology, Division of Reproductive Endocrinology and Infertility, and Florence Crozier Cobb Research Funds (to J J). McPherson Family Funds are also gratefully acknowledged.

## Author contribution statement

J J and K L conceived of the manuscript, contributed to its writing, and supervised revisions. S H-L and J R wrote and revised manuscript drafts, collected data, and summarized literature for tables. A A collected data and reviewed manuscript, L A and N L contributed to manuscript content and provided clinical context.
